# Extensive reshaping of bacterial operons by programmed mRNA decay

**DOI:** 10.1371/journal.pgen.1007354

**Published:** 2018-04-18

**Authors:** Daniel Dar, Rotem Sorek

**Affiliations:** Department of Molecular Genetics, Weizmann Institute of Science, Rehovot, Israel; Institut Pasteur, CNRS UMR 3525, FRANCE

## Abstract

Bacterial operons synchronize the expression of multiple genes by placing them under the control of a shared promoter. It was previously shown that polycistronic transcripts can undergo differential RNA decay, leaving some genes within the polycistron more stable than others, but the extent of regulation by differential mRNA decay or its evolutionary conservation remains unknown. Here, we find that a substantial fraction of *E*. *coli* genes display non-uniform mRNA stoichiometries despite being coded from the same operon. We further show that these altered operon stoichiometries are shaped post-transcriptionally by differential mRNA decay, which is regulated by RNA structures that protect specific regions in the transcript from degradation. These protective RNA structures are generally coded within the protein-coding regions of the regulated genes and are frequently evolutionarily conserved. Furthermore, we provide evidence that differences in ribosome densities across polycistronic transcript segments, together with the conserved structural RNA elements, play a major role in the differential decay process. Our results highlight a major role for differential mRNA decay in shaping bacterial transcriptomes.

## Author summary

Bacteria utilize operonic transcription to synchronize the expression of multiple consecutive genes. However, this strategy lacks the ability to fine-tune the expression of specific operon members, which is often biologically important. In this report, we integrate multiple transcriptome-wide RNA-sequencing methods to show that bacteria commonly employ differential mRNA decay rates for genes residing within the same operon, generating differential transcript abundances for equally transcribed operon members, at steady state. By comparing the transcriptomes of different bacteria, we show that differential decay not only regulates the expression levels of hundreds of genes but also often evolutionarily conserved, providing support for its biological importance. By mapping the RNA termini positions at steady-state, we show that stabilized operon segments are protected from different RNases through a combination of protective RNA structures, which surprisingly, are often encoded within protein-coding regions and are evolutionarily conserved. In addition, we provide evidence that differential ribosome densities over the regulated operons guide the initial events in the differential decay mechanism. Our results highlight differential mRNA decay as a major shaping force of bacterial transcriptomes and gene regulatory programs.

## Introduction

One of the defining features of bacterial gene expression is the co-transcription of multiple genes within polycistronic mRNAs [1]. Co-transcribed genes often encode proteins that participate in the same biological process or interact in a protein complex, allowing these genes to share the rate limiting steps of transcription. Thus, polycistronic transcription can synchronize the expression of functionally related genes, streamlining gene regulation in the compact genomes of bacteria.

A major limitation of polycistronic transcription is that it theoretically generates equal amounts of mRNA for all genes sharing an operon. This can be disadvantageous in the common scenario where one of the proteins encoded in the operon is needed in higher amounts than the others. In many such cases this inconsistency is resolved by differential translation efficiencies of individual genes encoded on the same transcript [2,3], or by internal transcription start sites (iTSSs) in the middle of the operon [4]. However, while differential translation efficiencies and iTSSs have been identified in many transcriptional units [2,3,5,6], differential operon expression patterns cannot always be explained by such mechanisms.

More than three decades ago it was shown that mRNA degradation can lead to differential mRNA stoichiometries for genes encoded on a single polycistron [7,8]. This regulation by differential mRNA decay was demonstrated for the *E*. *coli malEFG* operon encoding the maltose ABC-transporter complex, where MalF and MalG form the transmembrane channel and MalE acts as the substrate-binding protein, which is needed in higher quantities [8]. It was shown that the 3’ portion of the polycistronic mRNA (including *malFG*) is rapidly degraded by RNases whereas the 5’ portion (*malE*) is stabilized, leading to accumulation of the *malE* mRNA and to a substantial relative increase of the MalE protein as compared to MalF and MalG [8]. While regulation via differential operon decay was reported in multiple bacterial operons over the last few decades [9–17], the extent of this regulatory mechanism or its evolutionary conservation in bacteria remains unknown.

In this study, we combine a set of multi-layered high-resolution RNA-seq approaches to extensively map and characterize differentially decaying operons in *E*. *coli*. We find that regulation by differential decay is widespread and reshapes the stoichiometries of ~12% of polycistronic mRNAs in this model organism. This process is highly conserved also in the *Salmonella* and *Enterobacter* transcriptomes, and depends on conserved RNase-resistant RNA structures that guide the RNA decay dynamics. Furthermore, we find that differential decay is often dependent on translation and that stabilized operon segments are characterized by relatively higher ribosome densities than non-stabilized segments, suggesting that variation in translation efficiency guides endonuclease cleavage to ribosome depleted sections. Taken together, our data support differential operon decay as a common and frequently conserved mode of regulation in bacteria.

## Results

### Transcriptome-wide mapping of differentially decaying operons in *E*. *coli*

To examine the possible extent of differential RNA degradation in shaping operon stoichiometries, we first sought to limit our analysis to a set of well-defined *E*. *coli* operons annotated in the EcoCyc database [18]. We discarded from the set operons that were expressed at low levels or that are likely regulated by iTSSs or by internal partial transcription termination under the conditions tested in this study (Methods). For this, we comprehensively mapped the TSSs in exponentially growing *E*. *coli* bacteria using a previously established approach [18,19] (S1 Table), and excluded cases suspected as regulated by iTSSs (Methods). We further excluded cases where two consecutive genes within the operon were separated by an intrinsic terminator motif (a stem-loop structure followed by a uridine tract) or in which substantial Rho-dependent termination was previously described [20] (Methods). The final set included 390 significantly expressed multi-gene operons overall encompassing 1292 genes, representing 31% of the genes in the *E*. *coli* BW25113 genome (S2 Table).

**Fig 1:**
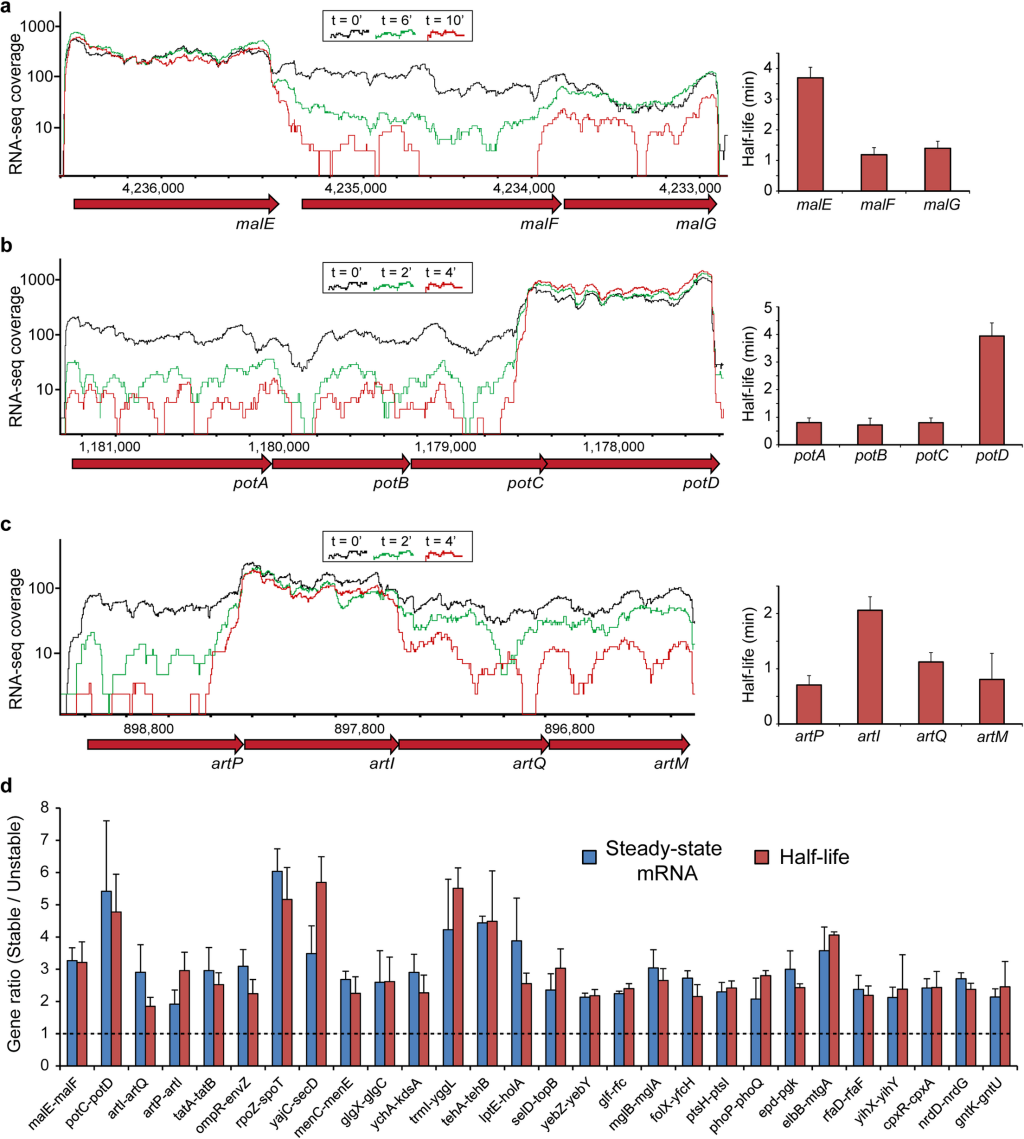
Identification of differentially decaying operons in *E*. *coli*. (A-C), Differential decay in three representative *E*. *coli* operons, depicted by normalized RNA-seq coverage in steady state (black, t = 0) or at two time points (green and red) following rifampicin treatment. RNA-seq coverage was normalized by the number of uniquely mapped reads in each library. Bar graphs show average half-life calculations from three replicates with error bars representing standard deviation. (D), Ratio of steady-state mRNA abundance (blue) and mRNA half-lives (red) shown for a subset of regulated gene-pairs in which mRNA abundances and decay rates closely matched. Gene names are marked below the x-axis. Shown is the average ratio between the genes with error bars denoting standard deviation.

We then treated exponentially growing *E*. *coli* cultures with rifampicin, a transcription initiation inhibitor [21], and tracked the RNA decay process by sequencing mRNAs collected at 1-minute intervals following transcription inhibition [22,23] (S1 Table; Methods). These data were used to calculate the half-life of individual genes (average half-life = 1.5 +/- 0.9 minutes), with the results overall agreeing well with recent RNA half-life calculations for *E*. *coli* genes [22,23] (S1 Fig; Pearson R = 0.75, *p < 10*^*−15*^; Methods). However, we detected 47 operons (12% of the 390 analyzed operons), encompassing 177 genes, in which two consecutive genes displayed correlated differential decay rates and differential steady-state mRNA levels (Fig 1A–1D; S3 Table; Methods). Re-analyzing RNA decay data from recent studies corroborated our results [22] (S3 Table).

It is well established mathematically and experimentally that the ratio in steady-state mRNA levels of two equally transcribed genes should equal to the ratio in their half-lives (for example, if the half-life of gene A is 2 minutes and the half-life of gene B is 1 minute, then at steady-state gene A will be 2-fold overexpressed as compared to gene B) [24]. Indeed, for the majority of operonic gene-pairs in which we found differential decay rates, the estimated differences in half-life closely matched the observed differences in steady-state mRNA abundance (Fig 1D; S3 Table). In about one quarter of the cases we found that the differences in decay rates did not fully match the differences observed in the mRNA level, indicating either measurement error or additional regulation by iTSSs or leaky termination in these operons (S3 Table).

Among the decay-regulated operons we identified was the previously described maltose ABC transport operon, *malEFG* [8] (Fig 1A). We measured the half-lives of the *malFG* and the *malE* segments to be 1 and 3.5 minutes, respectively, consistent with the relative stabilities measured for these genes in previous reports and validating our measurements. In addition to the *malEFG* operon we identified four other ABC transport systems, including those transporting polyamines, arginine, histidine and methionine (Fig 1B and 1C; S3 Table). In all these operons, we found that the stabilized gene was invariably the substrate-binding subunit, which is typically needed in higher quantities than the channel-forming units. This effect was independent of the gene’s position within the operon. For example, while the stabilized maltose substrate-binding subunit gene, *malE*, is the first gene in the operon (Fig 1A), the substrate-binding subunits of the polyamine (*potD*) and arginine (*artI*) transporters occur at the end or the middle of their operons, respectively (Fig 1B and 1C). Stabilization of the mRNA of the substrate-binding subunit results in higher steady-state mRNA levels of the respective gene, suggesting that differential decay is a common mechanism for tuning differential stoichiometries in ABC transport systems.

Another transport system subject to decay-based regulation was the *tatABC* operon, which encodes the evolutionarily conserved twin-arginine protein export system [25] (Fig 1D). In this system the TatA protein forms homo-polymeric ring structures that contain on average 25 TatA copies for each TatBC complex [25,26], and we indeed found the mRNA segment encoding the TatA subunit to be highly stabilized as compared to that encoding the TatBC subunits (S3 Fig).

We also observed differential decay in the *rpoZ-spoT* containing operon, such that the segment encoding *rpoZ* is stabilized compared with that containing *spoT*. The *rpoZ* gene encodes the RNAP omega subunit, which binds the regulatory alarmone ppGpp [27], and SpoT is one of the major enzymes involved in the synthesis and degradation of this alarmone [28] (S3 Table). As the enzymatic activity of a single SpoT rapidly converts multiple ppGpp molecules and hence can affect multiple *rpoZ* gene products, it is likely that RpoZ protein expression levels need to be higher than that of SpoT. Conceptually similar, we also identified differential decay in operons encoding 4 different two-component systems, where in each operon the transcription factor component was consistently more stable than the histidine-kinase enzyme [29]. Finally, we identified additional differentially decaying operons involved in cellular signaling and regulation, protein translocation across the membrane, antibiotic resistance and various metabolic processes, suggesting that differential decay plays an important role in post transcriptional regulation of many physiological processes in *E*. *coli* (S3 Table).

To examine whether regulation by differential decay is evolutionarily conserved we performed an identical rifampicin-based decay assay using the human pathogenic bacterium *Enterobacter aerogenes* grown under the exact same conditions as *E*. *coli* (S1 Table; Methods). We found that 71% (20/28) of decay-regulated orthologous operons expressed in *E*. *aerogenes* under the tested conditions show conserved differential decay patterns (S2 Fig; S4 Table). In addition, we compared the steady-state mRNA levels of *E*. *coli* decay-regulated operons with that of their orthologous operons annotated in *Salmonella typhimurium*, grown under the same conditions. This analysis showed that 68% (26/38) of the decay-regulated *E*. *coli* operons shared between the two species present similar patterns of sub-operon differential expression in *S*. *typhimurium* (S3 Fig; S1 and S5 Tables; Methods). The observed conservation of differential decay patterns between different bacteria highlights the functional importance of this phenomenon in bacterial gene regulation.

### Conserved internal RNA structures protect operon segments from decay

**Fig 2:**
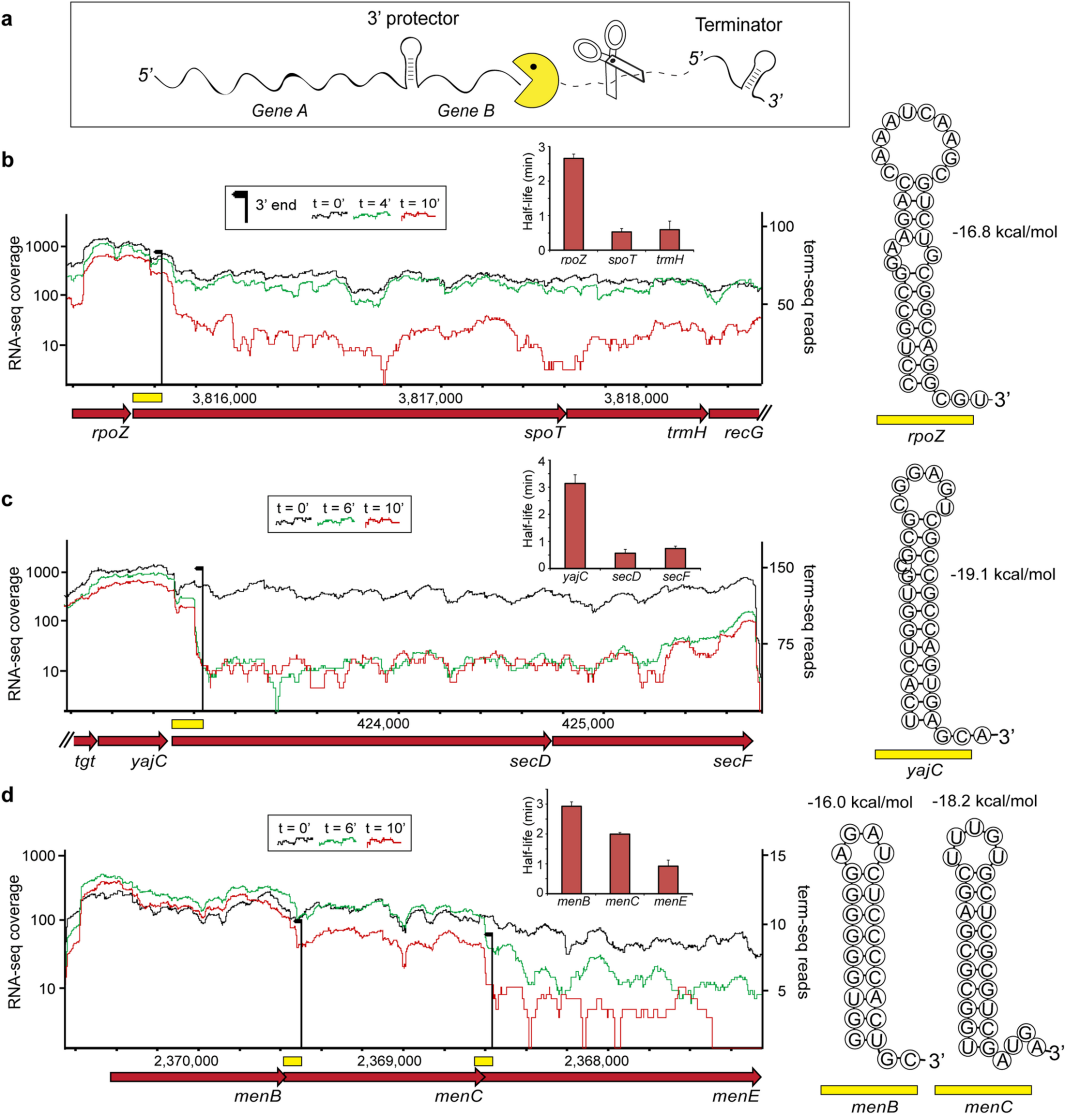
Protective RNA 3’ structures at the boundaries of differentially decaying transcript regions. (A), A model for differential decay in operons in which the 5’-most gene is preferentially stabilized, as described for the maltose operon [8]. Scissors and Pac-Man represent endonucleases and 3’-5’ exonucleases, respectively. (B-D), Differential decay in three representative *E*. *coli* operons, depicted by normalized RNA-seq coverage in steady state (black, t = 0) or at two time points (green and red) following rifampicin treatment. Bar graphs show average half-life calculations with error bars representing standard deviation. RNA-seq coverage was normalized by the number of uniquely mapped reads in each library. RNA 3’ ends detected by term-seq are shown as black arrows with the height of the arrow representing the total number of supporting reads. The sequences present immediately upstream to the recorded 3’ ends were folded using RNAfold [35] and are shown to the right of each coverage plot, with the estimated structure stability, measured in kcal/mol.

Our analyses discover many new cases where the steady-state levels of individual genes within polycistronic mRNAs are controlled via selective stabilization (differential operonic decay) (Fig 1; S3 Table). However, why some transcript regions are protected from digestion whereas others are rapidly degraded was unclear. In the well-studied maltose operon, it was shown that the *malE* gene is stabilized due to the presence of a 3’ protective RNA structure that resides in the intergenic region between *malE* and *malF*. This structure exerts its protective effect on *malE* following an initial endonucleolytic cleavage event that generates a functional *malE* fragment still physically attached to the protective element. This structure can then resists the 3’-5’ exonuclease processive activity typically performed in Gram negative bacteria by the Polynucleotide Phosphorylase (PNPase) and RNase II enzymes [30,31], thus stabilizing the *malE* gene, which is the 5’-most gene in the operon [8,32] (Fig 2A). Similar stabilization via 3’ RNA structures was also described in a few additional operons [9,10].

The set of differentially decaying operons we found included 34 gene-pairs (70%) in which the 5’-most gene is stabilized as compared with the downstream region, akin to the case of *malEFG* (Fig 1A; S3 Table). In 76% of these (26/34), the 3’ end of the stabilized mRNA portion (as determined by the term-seq method [33]) occurred immediately downstream to an energetically stable RNA-hairpin (Fig 2B–2D; S6 Table; Methods). These RNA structures were significantly more stable than those occurring randomly across the genome (S4 Fig; *p* < 10^−14^, Wilcoxon) and were not followed by the uridine tract required for Rho-independent termination [34] (S6 Table). In addition, we could not detect significant Rho-dependent termination at these sites using RNA-seq data from bicyclomycin-treated bacteria [20], supporting that these RNA structures have a role in 3’-5’ exonuclease resistance rather than transcription termination.

In the majority of cases (17/26), the structural RNA elements presumed to protect the upstream gene from degradation were embedded within the protein-coding sequence of the downstream gene, positioned on average 107 +/- 56 nt into the coding sequence (Fig 2B–2D; S6 Table). The sequence at the ORF of the downstream gene therefore carries, in addition to the protein-coding information, also the information guiding the differential decay of the transcript and thus, its stoichiometry at steady state. Examining the homologous genes in the *Salmonella* and *Enterobacter* genomes revealed that these protective RNA structures are conserved in 75% and 91% of the differential decay instances shared between *E*. *coli* and *Salmonella*, or *E*. *coli* and *Enterobacter*, respectively (S4 and S5 Tables). This correlated with an enhanced sequence conservation at the wobble codon positions that overlap protective RNA structures (*p* = 0.0008; S5 and S6 Figs; Methods). Interestingly, multiple protective structures can be observed in a single operon, generating complex decay patterns: for example, in the *menBCE* operon, we detected consecutive protective hairpins downstream of both the *menB* and *menC* genes, in correlation with the gradual decrease in stability and mRNA abundance detected in this operon (Fig 2D).

### RNase E-generated structured 5’ ends stabilize downstream operon regions

**Fig 3:**
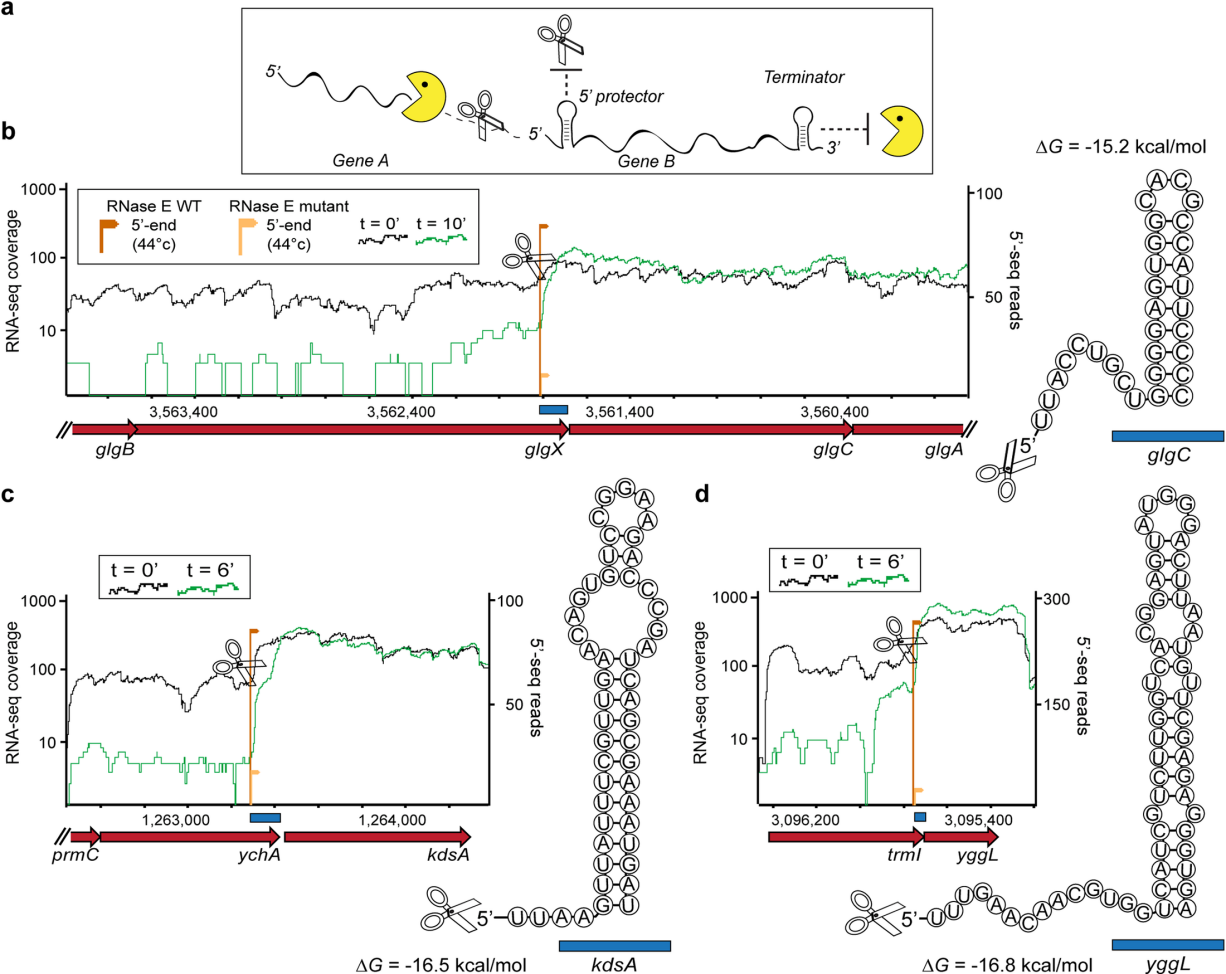
RNase E cleavage and protective 5’-end structures correlate with differential decay. (A), A model for differential decay in operons in which the middle or 3’-most gene is preferentially stabilized, as described for *papBA* [12]. Scissors and Pac-Man represent endonucleases and 3’-5’ exonucleases, respectively. (B-D), Differential decay in three representative *E*. *coli* operons, depicted by normalized RNA-seq coverage in steady state (black, t = 0) or an additional time points (green) following rifampicin treatment. RNA-seq coverage was normalized by the number of uniquely mapped reads in each library. The position and number of reads supporting RNase E cleavage sites in the WT strain or in the RNase E mutant are shown as dark and light orange arrows, respectively. The height of the arrows represents the total number of supporting 5’ end reads across all three replicates, normalized by the number of uniquely mapped reads in each experiment. The predicted structure and stability of the RNA sequence present immediately downstream of the RNase E cleavage site end is shown next to each gene, with blue rectangles specifying the position of the structure in the genome. The 5’ cleavage position is marked by a scissors cartoon.

In 30% of the decay-regulated operons we detected, the stabilized gene of the operon occurred in the middle of the transcript or was the most downstream one (e.g., Fig 1B and 1C; S3 Table). In these cases, protection from exoribonuclease activity cannot fully explain the observed stabilization, because no 5’-3’ exoribonucleases are known to exist in *E*. *coli* [30]. However, a similar downstream stabilization pattern was previously reported in the *papBA* operon of uropathogenic *E*. *coli*, encoding a transcription factor, PapB, and the major pilus protein, PapA [12]. It was found that the RNase E endoribonuclease cleaves the *papBA* mRNA at the intercistronic region separating the *papB* and *papA* genes, at a site located upstream of an RNA hairpin structure [36]. Consequentially, this processing event produces two transcript species: the unstable *papB* segment, which is rapidly degraded by 3’-5’ exonucleases, and the *papA* mRNA, which is protected by the 5’ RNA-structure from additional RNase E cleavage, as well as an RNase resistant terminator structure at its 3’ end [36] (Fig 3A).

To examine whether similar 5’-protective structures also occur in the decay-regulated operons described above, we conducted experiments with WT and a temperature-sensitive RNase E mutant, which is inactivated in the non-permissive temperature of 44°C [37,38] (Methods). We used 5’-end RNA sequencing to compare the repertoire of exposed mono-phosphorylated mRNA 5’-ends in the WT and mutant strains following brief incubation in 44°C, and identified RNase E cleavage sites as 5’-ends that were present in the WT but were consistently and substantially depleted in the inactivated RNase E mutant across three biological replicates (S1 Table; Methods). In 67% (10/15) of the operons described above, a clear RNAse E cleavage site was detected immediately upstream of the stabilized gene (Fig 3B–3D; S7 Table). All cleavage sites occurred in unstructured mRNA sections and at sequences closely matching the RNase E RN|WUU cleavage motif, with the conserved +2 uridine present in all cases [38] (Fig 3; S7 Table). Moreover, in 7 of these 10 operons, the cleavage site occurred closely upstream of a stable RNA structure, supporting a potential *papBA*-like protection mechanism in these cases (Fig 3B–3D). We found that all of the detected 5’ protective structures reside within the protein-coding region of the upstream unstable gene (Fig 3B–3D; S7 Table). For 80% (8/10) of these operons, an identical or closely positioned RNase E cleavage site, as well as protective structures, could be detected in *S*. *typhimurium*, based on recently published *in-vivo* cleavage maps, indicating a high degree of evolutionary conservation at the mechanistic level [38] (S7 Table).

### Differential decay is often dependent on translation and correlates with differences in ribosome density

While the RNA structures described above can insulate specific transcript regions during active degradation, the endonucleolytic cleavage events that initiate differential decay must first be directed to particular operon segments. For example, in the maltose operon, RNase E must preferentially cleave the mRNA encoding *malFG*, but not that of *malE*, such that the *malE* mRNA will remain physically connected to its protective RNA structure [8,32] (Fig 2A). However, the guiding factors that direct initializing cleavage events are poorly understood, even in the well characterized example of the *malEFG* operon [8,30,32].

**Fig 4:**
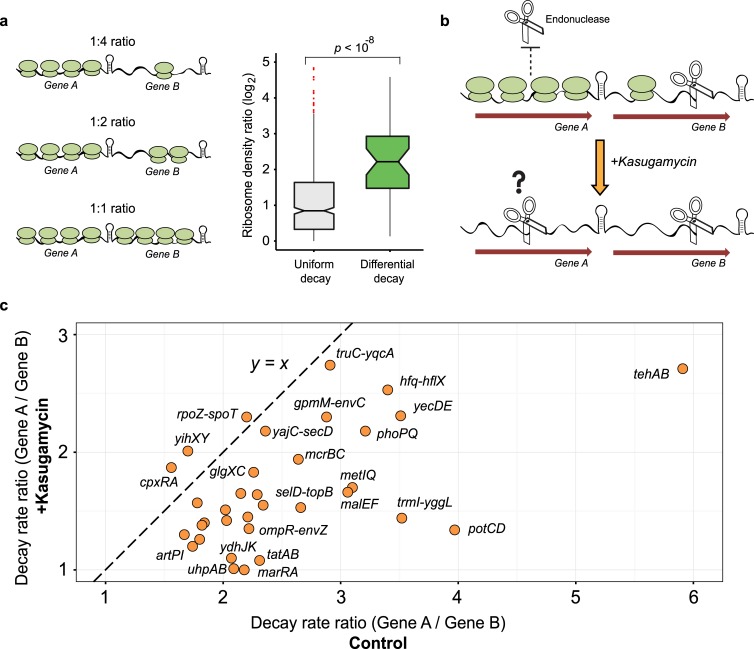
Ribosome density can guide differential operon decay. (A), Comparison of the relative ribosome densities across uniformly (n = 533) and differentially decaying operonic gene-pairs for which ribosome densities were previously measured (n = 39) [3] is shown as grey and green box-plots, respectively. Outliers are marked as red dots and the median is marked as a horizontal line within the box. The distributions were compared using a two-sided Wilcoxon rank-sum test (*p* < 10^−8^). On the left, a drawing illustrates the growing differences in ribosome densities between the genes along the boxplot’s y-axis. (B), Illustration of the effect of the translation-initiation inhibitor kasugamycin and its hypothesized effect on ribosome densities in polycistronic transcripts. (C), A scatter plot showing the change in relative decay rate of regulated gene-pairs calculated using recently published decay rates [22] for control (x-axis) and kasugamycin treated (y-axis) bacteria (S8 Table). The y = x function (denoting 1:1 ratio) is shown as a dashed line.

Ribosome densities were previously found to positively correlate with mRNA stability, presumably by physically restricting access to RNase cleavage sites [39–42]. Re-analyzing recently published ribosome profiling data [3], we found that in almost all differentially decaying gene-pairs the stabilized gene was covered by substantially more ribosomes than the non-stabilized genes in the operon (after normalization to transcript levels), with a median of 4.7-fold more ribosomes per transcript coating the stabilized mRNA segments (Fig 4A; Methods). Furthermore, such differential ribosome density profiles were significantly enriched in decay-regulated operons (Fig 4A; *p < 10*^*−8*^, Wilcoxon; Methods), implying that ribosome densities play a common role in shaping the differential decay process, possibly by decreasing endonucleolytic cleavage rates in operon regions populated by more ribosomes.

To directly examine whether differential ribosome density is involved in guiding differential decay, we analyzed recently published data in which *E*. *coli* mRNA half-lives were measured following a brief pre-exposure to kasugamycin, a translation initiation inhibitor [22]. In this experiment, kasugamycin treatment is expected to result in polycistronic transcripts where all genes are equally devoid of ribosomes, providing an approach to study the contribution of differential translation to operon decay (Fig 4B). Notably, the short inhibition of translation initiation resulted in a substantial reduction in differential decay in the vast majority of the regulated operons in our set (Fig 4C; S8 Table; Methods), providing evidence that differential decay within operons is often dependent on differences in translation efficiency.

**Fig 5:**
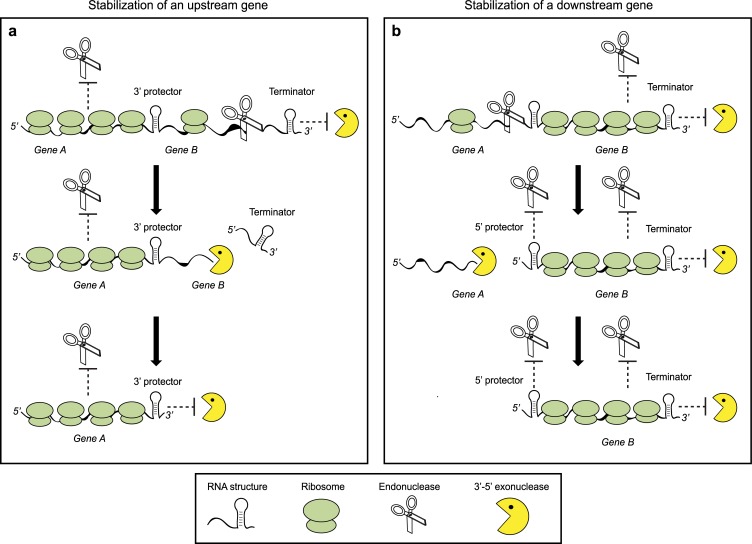
Proposed models for regulation of differential decay rates within the same transcript. (A), A model for stabilization of the upstream gene. The mature polycistronic transcript is protected from 3’-5’ exonucleases by the 3’ terminator hairpin structure. The relatively higher ribosome density over *gene A* provides protection from endonucleases, leading the RNase E to cleave the *gene B* segment, which results in the removal of the protective terminator structure, exposing *gene B* to rapid digestion by 3’-5’ exonucleases. *Gene A* remains protected by a 3’ RNA structure that blocks processive exonuclease activity. (B) A model for stabilization of the downstream gene. The mature operon is protected by the terminator structure. The relatively higher ribosome densities over *gene B* guide the initial cleavage to *gene A*. 3’-5’ exonucleases degrade *gene A* while *gene B* maintains its ribosome densities and protective terminator structure. Endonuclease cleavage occurs upstream of a protective 5’ structure that further protects the stable transcript from RNase E.

These results suggest that differences in ribosome densities guide the endonuclease cleavage events that initiate the differential decay process within polycistronic transcripts. Combined with the results from the above sections, we chart a general model for differential decay of polycistronic transcripts in *E*. *coli* (Fig 5).

## Discussion

Differential decay of polycistronic operons enables bacteria to reshape uniform transcription into differential expression. This process has been studied for the last 3 decades in multiple different species and operons, including in *malEFG* [8], *lacZYA* [15], *focA-pflB* [43], and *iscRSUA* [10] in *E*. *coli* K12; *papBA* [12] and *cfaAB* [11] in pathogenic *E*. *coli*; *pldB-yigL* in *Salmonella* [13]; and *pufBALMX* [9] in *Rhodobacter capsulatus*. In the current study, we took a transcriptome-wide approach to extensively map differentially decaying operons in *E*. *coli*. We find that differential decay is a common mode of regulation in bacteria, involved in re-shaping the stoichiometry of at least 12% of the *E*. *coli* operons, in a manner conserved between related species. We note that this number may actually be higher as recent studies reported the existence of condition specific differential decay [10,13]. The differential decay data produced in this study were organized into an interactive online browser that is available at: http://www.weizmann.ac.il/molgen/Sorek/operon_decay/.

We show, using a combination of 3’ and 5’ RNA-termini sequencing, that protective RNA structures occur at the boundaries of stabilized operon segments in the vast majority of cases and are generally encoded within the protein-coding regions of the flanking, unstable genes. In addition, we find that the less stable genes in the operon are covered by fewer ribosomes per transcript and that translation plays a major role in shaping the differential decay process. While the relation between ribosomes and mRNA decay has been well-established for monocistronic mRNAs [39–41,44], our results extend this concept to multi-gene operons with differentially decaying transcript segments. Importantly, these observations provide a potential explanation for how specific operon segments are selected for initial endonucleolytic cleavage by RNases with degenerate target motifs, a piece of the mechanism that was so far less understood (Fig 5). Interestingly, stable structures at protein-coding mRNA regions were previously suggested to reduce translation efficiency [44–46], implying that protective structures could actually play a dual role: first, blocking translation initiation, which reduces ribosome density on the flanking gene and exposes the region to increased RNase E dependent cleavage, and second, direct protection of the stable transcript region from the decay process.

Although our differential decay models provide a potential mechanism for most of the regulated operons in our dataset, additional factors, such as trans-acting ncRNAs, have been found to play a role in shaping operon decay patterns by modulating access to rate-limiting endonuclease cleavage sites [10,13]. Notably, such ncRNAs enable condition-specific stoichiometric regulation in operons that are otherwise degraded uniformly. Indeed, our analysis failed to detect differential decay in both the *iscRSUA* and the *pldB-yigL* operons, which were recently shown to be regulated by ncRNAs activated under conditions other than the ones employed in our study [10,13]. Thus, considering the large number of trans-acting ncRNAs and antisense RNAs in bacteria, the extent of differential decay-based regulation is likely even greater than our current estimates.

Whereas our proposed models for differential decay likely hold for other organisms that share similar RNA decay machineries (especially proteobacteria that rely on RNase E and 3’-to-5’ exonucleases similar to *E*. *coli*), many bacterial lineages harbor different RNase combinations and properties, for example 5’-3’ processive exonucleases in Firmicutes [30]. Presumably, differential decay in such organisms may rely on different principles or additional molecular signals.

## Methods

### Strains growth conditions and RNA extractions

*Escherichia coli* BW25113, *Salmonella enterica subsp*. *enterica serovar Typhimurium SL1344* and *Enterobacter aerogenes* KCTC 2190 were cultured in LB media (10g/L tryptone, 5g/L yeast extract 5g/L NaCl) under aerobic conditions at 37°C with shaking to an optical density (OD_600_) of 0.5. Prior to sample collection, 1:10 ice-cold stop solution (90% ethanol and 10% saturated phenol) was added and the cultures were immediately placed on ice to stop all cellular processes [22,47]. Bacterial pellets were collected by centrifugation (4000 rpm, 5 min, at 4°C); flash frozen and stored in -80°C until RNA extraction. For RNA isolation, frozen pellets were thoroughly resuspended and mixed in 100μl lysozyme solution (3mg/ml in 10mM Tris-HCl and 1mM EDTA) pre-warmed to 37°C and then incubated at 37°C for 1min. The cells were then lysed by immediately adding 1ml tri-reagent (Trizol) followed by vigorous vortexing for 10s until solution is cleared. Following an incubation period of 5min at room temp (RT), 200μl chloroform was added and the sample was vortexed for another 10s until homogeneous. The sample was incubated for 2-5min at RT until visible phase separation was observed and then centrifuged at 12,000g for 10min. The upper phase was gently collected (about 600μl) and mixed at a 1:1 ratio with 100% isopropanol and then mixed by vortexing for 2-3s. The sample was incubated for 1h at -20°C and then centrifuged (14,000rpm, 30min, at 4°C) to collect the RNA pellet. The solution was removed without disturbing the pellet, followed by two consecutive wash rounds using 750μl 70% ethanol. The pellets were air dried for 5min and then dissolved in nuclease free H_2_0 and incubated for 5min at 50°C. All RNA samples were treated with TURBO deoxyribonuclease (DNase) (Life technologies, AM2238).

### RNA sequencing, read mapping and TSS mapping

RNA-seq, term-seq and 5’-sequencing libraries were prepared and sequenced as previously described [33,48]. Sequencing was performed using the Illumina NextSeq 500 and the data was deposited in the European Nucleotide Database (ENA) under accession no. PRJEB21982 (S1 Table). Sequencing reads generated for *E*. *coli*, *S*. *typhimurium* and *E*. *aerogenes* were mapped to the CP009273, NC_016810.1 and NC_015663.1 RefSeq genomes, respectively, using NovoAlign (Novocraft) V3.02.02 with default parameters, discarding reads that were non-uniquely mapped as previously described [33]. For 5′-end sequencing, the RNA was divided into a tobacco acid pyrophosphatase (TAP)–treated and untreated (noTAP) reactions to enable primary transcript detection and then sequenced using 5’-seq [33,49]. TSSs were mapped as was recently described [19].

### Transcriptome-wide RNA decay analysis

*E*. *coli* or *E*. *aerogenes* overnight cultures were diluted 1:100 into 25ml fresh LB media and incubated at 37°C until reaching an optical density (OD_600_) of 0.5. The culture was then placed in a preheated 37°C shallow water bath to preserve the experiment temperature and 125μl Rifampicin (100mg/ml, for a final concentration of 500μg/ml) were immediately added to the culture to inhibit RNA synthesis. Selected time points were sampled by collecting 1.4ml from the culture into a pre-chilled tube containing 170ul of ice-cold stop solution (90% ethanol and 10% saturated phenol) to deactivate cellular processes and RNA-decay. The sample was quickly vortexed and then placed on ice until all time points were collected. The samples were centrifuged for 5min at 4000rpm to collect cell pellets and were flash frozen. During RNA extraction, after tri-reagent mediated lysis, each sample was spiked with 5fmol of the ERCC RNA (Ambion, 4456740) to allow normalization of RNA abundance between samples. RNA-seq libraries were prepared and sequenced as described above. Gene-expression was calculated as the median coverage per nucleotide (reads/nt) normalized by the number of reads that mapped to all ERCC spike-in RNA, an estimate which we found is more robust than the total number of reads or average coverage in cases of non-uniform decay patterns, where overlapping operon regions, or small sub-ORF regions can persist long after the full transcript has been eliminated. Transcript half-lives were calculated by fitting the decay time-course abundance measurements per gene with a delayed exponential-decay function as previously described [22]. The previously published half-lives for the 779 transcripts described in S1 Fig were taken from the “Fig 4 Source data 1” in [22], which provides the estimated Decay rate (λ) per gene. To extract the half-life we calculated t_1/2_ = ln(2)/ λ.

### Operon selection and gene-pair analysis

Operon gene annotations were extracted from EcoCyc [18] (S2 Table). To identify and analyze gene-pairs found within the same transcriptional unit, consecutive gene-pairs were only considered if the following criteria were met: i) the intergenic region interspacing the genes was shorter than 200nt. ii) The downstream gene was not associated with an independent TSS under the growth conditions of this study [19]. iii) The upstream gene was not associated with a term-seq site displaying an intrinsic terminator signature (i.e., hairpin followed by a uridine stretch). iv) No substantial rho-dependent termination measured in *E*. *coli* treated with the Rho inhibitor Bicyclomycin [20]. v) The stable and unstable genes were covered by median greater than or equal to 10 and 1 reads/nt, respectively. vi) The expression difference between the genes was not greater than 10-fold, as we find such high values were sometimes indicative of incorrect operon annotation or highly active secondary promoters. vii) The decay rate of both genes was measured in at least one biological replicate of the experiment.

Gene-pairs in which one gene was at least 2-fold more stable and 2-fold more abundant than its consecutive neighbor gene were classified as putatively decay-regulated (S3 Table). We manually accepted 4 gene-pairs displaying borderline, yet consistent signal as well as 7 differentially expressed gene-pairs in which the decay rate was not measured in our experiment usually due to lack of expression in the conditions tested, but for which differential decay could clearly be identified in a recently published dataset [22] (S3 Table).

### Detection of internal protective RNA elements using term-seq

Term-seq libraries were prepared as previously described [33] and sequenced using a paired-end sequencing approach [48] (S1 Table). The number of 3’-end reads per genomic position was calculated and for each 3′ site the average library insert length was calculated using the paired-end read mapping positions. Sites were then associated with their respective genes, requiring that the average insert length would overlap the gene coding region [48]. The position supported by the highest number of reads associated with a stabilized gene was selected as the representative 3’-terminus of the stabilized RNA in steady-state (S4 and S6 Tables). In a few cases, a different, slightly less covered position was selected instead if it provided a substantially better fit for the decay pattern observed. The sequence upstream of each selected site was extracted from the genome and folded using RNAfold [35] to evaluate the predicted structure and its estimated stability (kcal/mol) (S4 and S6 Tables).

### Comparative transcriptomic analysis of differentially decaying operons

The *S*. *typhimurium* and *E*. *aerogenes* protein-coding sequences were retrieved from NCBI and blasted against the *E*. *coli* protein database, with E-value set to 10^−5^. Gene and operon orthologues were classified as the Best Bi-directional Hits (BBHs). Gene-pairs were compared if they occurred consecutively as in *E*. *coli* and were substantially expressed, as described above (S4 and S5 Tables).

### Evaluation of conservation rates at codon positions that overlap protective structures

Genes containing a protective structure embedded at least 50 bases into the coding region were selected (n = 23) and their orthologues from up to 21 different bacterial strains belonging to the Enterobacteriaceae family (including the *E*. *aerogenes* and *S*. *typhimurium* strains in this study) were identified using blast (S9 Table). Gene information for each organism was downloaded from the Integrated Microbial Genomes (IMG) database [50]. Gene orthologues were discarded if the gene sizes differed by more than 50 bases. Genes were then aligned using Clustalw2 [51] and the conservation at each position in the alignment was defined as the maximal base frequency detected at that position. Conservation was calculated for each of the codon positions independently (as shown in S5 Fig). The average conservation at positions overlapping protective structures was calculated using a window size of 50 bases. In cases where the stabilizing structure occurred at the end of the gene (as in Fig 3) the window was defined as 70 bases to accommodate the longer structures found in these genes. The conservation in control gene regions that do not contain a known protective structure was calculated using a sliding window approach (using the same window size and sliding each window by 5 codons at a time over the entire protein coding region).

### Detection of RNase E-depended cleavage sites upstream of stabilized operon regions

WT *E*. *coli* and temperature-sensitive RNase E mutants were generously provided by the McDowall lab [37]. Triplicates of each strain were grown in the permissive temperature of 30°C overnight in LB and were diluted the next day 1:100 in fresh media. The cultures were then grown in 30°C until reaching an OD of 0.5 and were then incubated at 44°C for 10min to briefly deactivate RNase E in the mutant but not the WT. The cells were collected and the RNA was extracted as described above. The mono-phosphorylated 5’ends were sequenced using 5’-end sequencing (noTAP only, as described above). Putative RNase E cleavage positions, tightly matching the decay pattern observed with RNA-seq, were considered if they covered by a sum of at least 20 reads across all replicates and showed >2.5-fold average enrichment in the WT compared with the mutant samples. In cases where multiple potential cleavage sites were available, the most highly covered site was selected. The sequence downstream of the RNase E cleavage site was analyzed and folded using RNAfold [35] (S7 Table).

### Differential ribosome density across uniformly and differentially decaying operons

Normalized ribosome densities (ribosome counts per mRNA) were retrieved from a recently published dataset [3]. The ribosome density ratio between consecutive operon gene members was calculated for all analyzed gene-pairs (S3 Table) in which translation efficiency could be calculated for both genes in the dataset [3]. The differential translation distributions of uniformly (n = 533) and differentially decaying (n = 39) gene-pairs were then compared using a two-sided Wilcoxon rank-sum test.

### The effect of inhibiting translation initiation on relative decay

Estimated decay rate values were retrieved from a recently published dataset, in which *E*. *coli* bacteria were pre-exposed to 1mg/ml kasugamycin for 15min before being subjected to a transcriptome-wide rifampicin-based RNA decay assay [22]. The decay ratio for the control and kasugamycin-treated samples was calculated in all cases where such values were available for both genes in at least one replicate. In cases where data was available for the two published replicates, the average decay ratio was used instead.

## Supporting information

S1 Table Library description and data deposition accession numbers in ENA. (XLSX)

S2 Table *E*. *coli* operons used in study. (XLSX)

S3 Table Differentially decaying operons in *E*. *coli*. (XLSX)

S4 Table Conserved differentially decaying operons and term-seq sites in *E*. *aerogenes*. (XLSX)

S5 Table Conserved differentially expressed operons and term-seq sites in *S*. *typhimurium*. (XLSX)

S6 Table *E*. *coli* term-seq sites in regulated operons with sequence and structure. (XLSX)

S7 Table RNase E sites in regulated operons with sequence and structure in *E*. *coli* and *S*. *typhimurium*. (XLSX)

S8 Table The effects of kasugamycin on gene-pair decay ratios. (XLSX)

S9 Table Bacteria used for conservation analysis. (XLSX)

S1 Fig Correlation of half-lives measured in this study with previously published data. Comparison of decay rates calculated in this study (x-axis) and recently published values (y-axis) [22]. The scatter shows genes for which a half-life was measured in both datasets (n = 779). Pearson correlation value is shown above (R = 0.75).
(EPS)

S2 Fig Conservation of differential operon decay between bacteria. Comparative RNA-decay analysis in *E*. *coli* and *E*. *aerogenes* depicted by normalized RNA-seq coverage in steady state (black, t = 0) or at two time points (green and red) following rifampicin treatment. RNA-seq coverage was normalized by the number of uniquely mapped reads in each library. RNA 3’ ends detected by term-seq are shown as black arrows, with the height of the arrow representing the total number of supporting reads. (A), The *ptsHI-crr* operon shows a conserved decay signature and 3’ end processing patterns in *E*. *coli* and *E*. *aerogenes*. (B-C), Conserved RNA decay patterns detected in two additional operons encoding the *hfq-hflX* and *bepA-yfgD* gene-pairs.
(TIF)

S3 Fig Conserved differential expression of sub-operonic segments in *E*. *coli* and *S*. *typhimurium*. (A), Ratio of steady-state mRNA abundance of consecutive decay-regulated gene-pairs in *E*. *coli* (blue) and *Salmonella typhimurium* (green) as measured by RNA-seq. Average of three biological replicates is shown with error bars representing standard deviation. (B-C), Examples of differential mRNA abundance in conserved *E*. *coli* and *S*. *typhimurium* operons with the median RNA-seq coverage/nt, calculated from a representative replicate, shown above the genes in red.
(TIF)

S4 Fig Structures of protective 3’ end RNA elements. The 45 nucleotide sequence occurring immediately upstream of the recorded 3’ ends present at the sites of decay, as detected by term-seq (n = 26; Methods), as well as randomly selected intergenic genomic positions (n = 10,000), were folded using RNAfold [35] and the RNA-structure stability distributions were plotted. Outliers are depicted as red dots. The distributions were compared using a two-sided Wilcoxon rank-sum test (*p* < 10^−14^).
(EPS)

S5 Fig Increased sequence conservation at wobble positions in gene regions containing a protective RNA structure. Enhanced sequence conservation at the wobble codon positions of genes encoding a protective RNA structures (n = 23). The nucleotide sequence of genes encoding a protective structure (n = 23) was compared among up to 21 different bacteria (S9 Table; Methods). The boxplot shows the conservation level at the three codon positions, independently, for gene regions that contain a protective structure (red boxes) and regions of the same size that do not contain a structure (grey boxes). The distributions were compared using a two-sided Wilcoxon rank-sum test and the p-values are reported in the Fig.
(EPS)

S6 Fig Examples of increased conservation within gene regions containing a protective RNA structure. Multiple sequence alignments generated for three representative genes that contain a protective structure. Rows show sequences from different organisms as noted and the Integrated Microbial Genomes (IMG) genome identification number for each organism is listed. A black box shows the region containing the protective RNA structure and the predicted RNA fold is shown above. Red stars represent positions with 100% sequence conservation.
(EPS)
