# Supplementary figures and images for: Extensive reshaping of bacterial operons by programmed mRNA decay
Source: PLoS Genet. 2018 Apr 18;14(4):e1007354. doi: 10.1371/journal.pgen.1007354 (PMC5927463; doi:10.1371/journal.pgen.1007354)

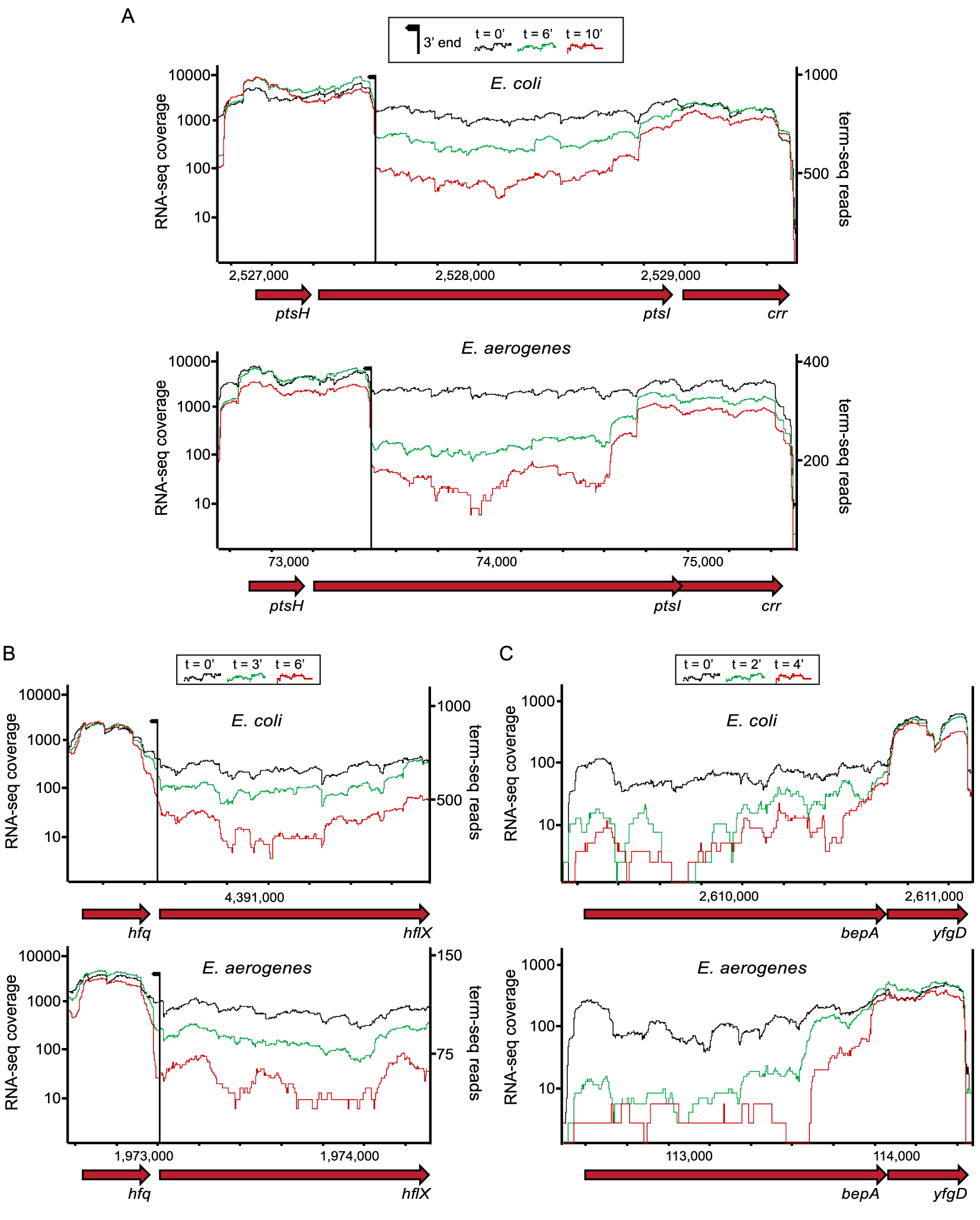

Supplement: S2 Fig — Comparative RNA-decay analysis in E. coli and E. aerogenes depicted by normalized RNA-seq coverage in steady state (black, t = 0) or at two time points (green and red) following rifampicin treatment. RNA-seq coverage was normalized by the number of uniquely mapped reads in each library. RNA 3’ ends detected by term-seq are shown as black arrows, with the height of the arrow representing the total number of supporting reads. (A), The ptsHI-crr operon shows a conserved decay signature and 3’ end processing patterns in E. coli and E. aerogenes. (B-C), Conserved RNA decay patterns detected in two additional operons encoding the hfq-hflX and bepA-yfgD gene-pairs. (TIF) [file pgen.1007354.s011.tif]

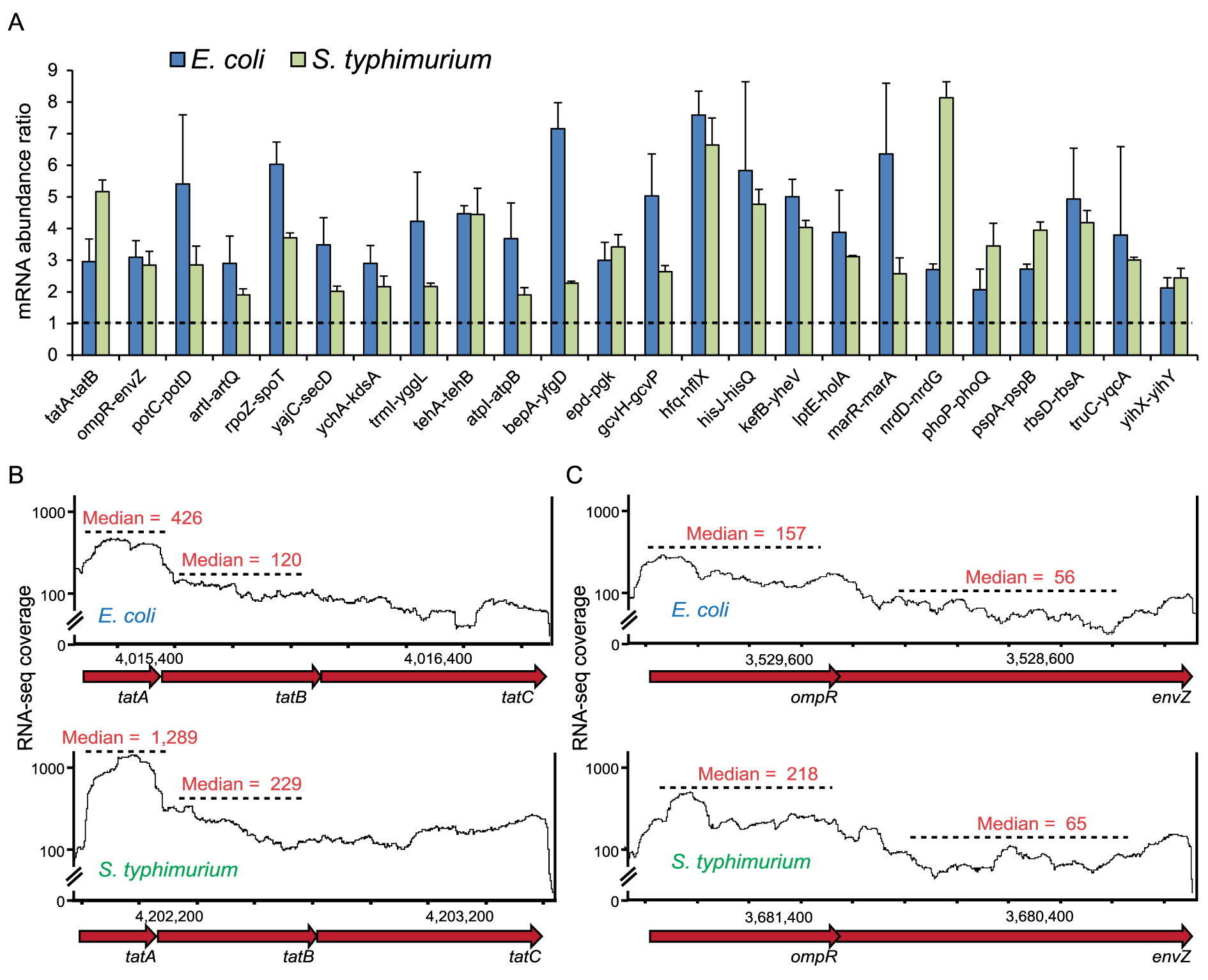

Supplement: S3 Fig — (A), Ratio of steady-state mRNA abundance of consecutive decay-regulated gene-pairs in E. coli (blue) and Salmonella typhimurium (green) as measured by RNA-seq. Average of three biological replicates is shown with error bars representing standard deviation. (B-C), Examples of differential mRNA abundance in conserved E. coli and S. typhimurium operons with the median RNA-seq coverage/nt, calculated from a representative replicate, shown above the genes in red. (TIF) [file pgen.1007354.s012.tif]
